# [Retracted] Breast cancer cells are arrested at different phases of the cell cycle following the re-expression of ARHI

**DOI:** 10.3892/or.2025.8916

**Published:** 2025-05-19

**Authors:** Xiaoxiao Zuo, Yan Qin, Xiaojin Zhang, Qian Ning, Shan Shao, Minna Luo, Na Yuan, Shangke Huang, Xinhan Zhao

Oncol Rep 31: 2358–2364, 2014; DOI: 10.3892/or.2014.3107

Subsequently to the publication of the above article, a concerned reader drew the Editor's attention to the fact that, comparing the western blot data shown in Fig. 2 on p. 2360 and Figs. 5 and 6 on p. 2363, two of the β-actin control protein bands appeared to be shared in common between Figs. 2 and 6, and one of the P21 protein bands in Fig. 6 was strikingly similar to a Cyclin D1 protein band shown in Fig. 5. Furthermore, based on the appearance of the gels, there appeared to be several breaks in the continuity of the gel slices between the second and third lanes in Fig. 6, such that these data were apparently not derived from the same set of experiments.

Upon consulting the authors in relation to the unusual features in the abovementioned western blots in this paper, they were unable to offer a satisfactory explanation to account for the presentation of these figures (although offering to repeat the experiments in question). In view of the uncertainties in the affected data, the Editor of *Oncology Reports* has decided that this paper should be retracted from the Journal on account of a lack of confidence in the data. The authors were asked for an explanation to account for these concerns, but the Editorial Office did not receive a reply. The Editor apologizes for any inconvenience caused.

